# Association between vitamin D deficiency and the risk of prevalent type 2 diabetes and incident prediabetes: A prospective cohort study using data from The Irish Longitudinal Study on Ageing (TILDA)

**DOI:** 10.1016/j.eclinm.2022.101654

**Published:** 2022-09-17

**Authors:** Kevin McCarthy, Eamon Laird, Aisling M. O'Halloran, Cathal Walsh, Martin Healy, Annette L. Fitzpatrick, James B. Walsh, Belinda Hernández, Padraic Fallon, Anne M. Molloy, Rose Anne Kenny

**Affiliations:** a The Irish Longitudinal Study on Ageing, Trinity College Dublin, Dublin, Ireland; b School of Medicine, Trinity College Dublin, Dublin, Ireland; c Department of Mathematics and Statistics, University of Limerick, Limerick, Ireland; d Biochemistry Department, Laboratory Medicine & Molecular Pathology, St James's Hospital, Dublin, Ireland; e Departments of Family Medicine, Epidemiology, and Global Health, Schools of Medicine and Public Health, University of Washington, Seattle, Washington, United States of America

**Keywords:** Diabetes, Prediabetes, Vitamin D, TILDA, Public health

## Abstract

**Background:**

It is hypothesized that vitamin D contributes to the aetiology of type 2 diabetes mellitus (diabetes). This study's objective was to examine the relationships between baseline vitamin D status (as measured by plasma 25-hydroxyvitamin D concentration) and both prevalent diabetes and prospective risk of developing diabetes, including prediabetes, in a population with historically low levels of vitamin D.

**Methods:**

In this prospective cohort study, data from The Irish Longitudinal Study on Ageing (TILDA), a nationally representative cohort of adults aged ≥50 years residing in Ireland were analysed, including wave 1 (October 2009–June 2011) (*n* = 5272) and wave 3 (March 2014–October 2015) (*n* = 3828). Those aged <50 years at baseline or who did not complete the health assessment were excluded. Logistic regression models examined the associations between baseline vitamin D concentration (nmol/L) with prevalent diabetes status and incident diabetes/prediabetes collected at a 4-year follow-up. Models were adjusted for age, sex, education, body mass index, smoking history, physical activity, use of statins, and the season in which the vitamin D concentration was sampled.

**Findings:**

Deficient baseline vitamin D concentration was cross-sectionally associated with an increased likelihood of having prevalent diabetes (Relative Risk Ratio [RRR] 1·5, 95% CI: 1·03, 2·18; *p* = 0·037). In longitudinal analyses evaluating diabetes status 4 years later, there was a 62% increased likelihood (RRR: 1·62, 95% CI: 1·12, 2·35; *p* = 0·011) of developing prediabetes for those with vitamin D <30 nmol/L compared to those with ≥75 nmol/L. The rate of progression from prediabetes to diabetes between wave 1 and 3 was observed to be 32·5%.

**Interpretation:**

Those with lower concentrations of vitamin D, as measured by 25-hydroxyvitamin D, may have different risk profiles with regards to their glycaemic status. Our study had limited power due to the low incidence of diabetes but showed strong associations with incident prediabetes, so further research is required. Optimising vitamin D status at a population level may significantly reduce diabetes.

**Funding:**

TILDA is funded by Atlantic Philanthropies, the Irish Department of Health, and Irish Life, while additional funding was provided by the Irish Department of Agriculture, Food and the Marine (13F492) to cover the cost of 25-hydroxyvitamin D analysis.

**Research in context**

**Evidence before this study**

We reviewed the PubMed database from 1^st^ January 2001 to 31^st^ December 2021 using the terms “diabetes mellitus” or “type 2 diabetes” or “insulin resistance” and “vitamin D” or “25(OH)D and “association” and “human”, excluding articles that were not reporting results from an observational study, clinical trial, or randomized control trial. We observed numerous studies reporting associations between vitamin D and insulin resistance and/or diabetes mellitus. The reported evidence on the associations were inconsistent with studies using different criteria, doses, baseline vitamin D concentrations above sufficient status, and lack of adjustment for important co-variates. We hypothesised that vitamin D deficiency increased the likelihood of developing both prediabetes and diabetes given the spectral nature of glycaemia and the underlying biological processes involved.

**Added value of this study**

We were able to test our hypothesis in a large population-based study, with nearly 4000 participants and a 4-year follow-up period, where the population had low baseline vitamin D levels in a country with no mandatory food fortification. Using cut-off points for vitamin D, accepted by clinicians with regards to bone health, we observed that vitamin D deficiency (<30 nmol/L) was associated with a 62% increased likelihood of developing prediabetes when compared to those with baseline levels ≥75 nmol/L.

**Implications of all the available evidence**

Our findings support the hypothesis that poor vitamin D status contributes to the aetiology of type 2 diabetes mellitus and warrant consideration in the design of potential interventional studies which may ultimately influence clinical practice and/or public health policy. While the effect size shown here may be small, health and economic impact at the population level could be significant, in an ageing society with an increasing burden of diabetes prevalence and associated comorbidities.

Alt-text: Unlabelled box

## Introduction

Type 2 diabetes mellitus (diabetes) is a multifactorial disease involving genetic and environmental factors. It is characterised by dysregulation of carbohydrate, lipid, and protein metabolism due to impaired insulin secretion, increased insulin resistance, or a combination of both, resulting in hyperglycaemia and chronic inflammation.^1^ Diabetes causes both micro and macrovascular complications, leading to increased rates of ischaemic heart disease, chronic kidney disease, cognitive decline, and visual impairment among other conditions. Risk factors for diabetes include increasing age, central obesity, and physical inactivity.^2^ In Ireland, the prevalence of diabetes is almost 10% in those ≥50 years, with nearly one in ten of those being undiagnosed.^3^ Diabetes is becoming an increasingly prevalent condition,^4^ and is a leading cause of disability and death,^5^ estimated to affect one in twelve adults worldwide and projected to increase by 55% by 2035.^6^

Vitamin D is a seco-steroid hormone that is essential for bone and musculoskeletal health through increasing intestinal absorption of calcium.^7^ Vitamin D receptors (VDR) are expressed in many non-skeletal cells, and it is believed vitamin D may have numerous extra-skeletal effects. Vitamin D has been associated with immune responses, cardiovascular disease, diabetes, and cancer in observational studies.^8^ The main source of vitamin D is skin synthesis following exposure to ultraviolet B (UVB) light. In many far latitude countries, including Ireland (51-55°N), this is limited to the summer months, with a sole reliance on dietary intake during the winter period, if not year-round, depending on people's lifestyle and sun exposure. Importantly, few foods contain sufficient vitamin D and these are infrequently consumed. Current estimates of vitamin D deficiency (<30 nmol/L) within Irish adults ≥50 years is 1 in 8, which increases to nearly 1 in 2 for those aged >85 years despite a prevalence of vitamin D supplement use of 8·5%.^9^

In the context of the development of diabetes, there are numerous molecular mechanisms that support vitamin D having a role in glucose homeostasis, from the ability of pancreatic beta cells to convert inactive vitamin D to its active metabolite, a process that is necessary for insulin secretion, to vitamin D influencing the expression of genes that play a role in insulin signal transduction.^10^, ^11^, ^12^ Low levels of vitamin D have been hypothesized to increase the risk of insulin resistance and the development of diabetes, through effects on hepatic glucose and lipid metabolism as well as pancreatic islet cell function and survival. ^13^

The aim of this paper was to examine the cross-sectional and longitudinal relationship between baseline vitamin D status, as measured by 25-hydroxyvitamin D (25(OH)D) concentration, and risk of developing diabetes in an older Irish population in both those with normoglycaemia and prediabetes.

## Methods

### Sample

This study utilised data from the first and third waves of The Irish Longitudinal Study on Ageing (TILDA), a prospective study of the health, social and economic circumstances of community-dwelling adults aged ≥ 50 years in Ireland. The data in the first wave (baseline) was collected between October 2009 and June 2011, while the third wave was collected between March 2014 and October 2015. The data collection process has been described in detail elsewhere.^14^ As part of the study participants completed a Computer Assisted Personal Interview (CAPI), conducted in participants' homes by trained interviewers with detailed questions relating to socio-demographic characteristics and health at each wave. Participants were also invited to take part in a health assessment, carried out in a dedicated centre by trained research nurses, at both waves 1 and 3. This assessment included anthropometric, cognitive, and cardiovascular tests, as well as blood draws. A modified home-based assessment was offered if participants were unable or unwilling to travel to a health centre. TILDA health assessments were carried out throughout the day, with some participants undergoing their health assessment in the morning and others in the afternoon so these measurements were not made at a standardized time of day.

The inclusion criteria for these analyses required participants to be age ≥50 years at wave 1, having participated in both wave 1 and wave 3, and having completed blood collection resulting in laboratory values for Vitamin D at wave 1 as well as health assessment and HbA1c, a measurement of glycated haemoglobin, at both waves (Supplemental Figure 1).

### Diabetes status

The methodology for HbA1c analysis at wave 1 in TILDA has been described previously.^3^ Briefly, 10 ml of fresh whole blood was collected in EDTA‐coated tubes and transported to a central processing laboratory in temperature‐controlled shipping boxes that maintained the samples at 2–8°C for up to 48 hours. Buffy coat samples were then isolated in 1 ml aliquots and placed in long‐term storage at −80°C before subsequent thawing for analysis. HbA1c concentration was analysed by reversed‐phase cation‐exchange chromatography. At wave 3, HbA1c analysis was done using the same methods other than use of frozen whole blood samples rather than buffy coat samples. Validation of the measurement of HbA1c from buffy coat samples and correlation between whole blood and buffy coat samples has been described previously.^3^

At both wave 1 and wave 3, diabetes was identified by the presence of any of the following criteria: a self-reported doctor diagnosis of diabetes, use of diabetes medication as identified by using the World Health Organisation Anatomic Therapeutic Classification codes (A10A for insulin and A10B for non-insulin hypoglycaemics) or a HbA1c level ≥48 mmol/mol as per the American Diabetes Association criteria.^2^

At both wave 1 and wave 3 prediabetes was identified by those who had a HbA1c ≥39 mmol/mol and <48 mmol/mol, as per the American Diabetes Association criteria,^2^ but who were not taking any diabetes medications and did not have a self-reported doctor's diagnosis of diabetes.

Those participants who reported a doctor diagnosis of diabetes age ≤40 at wave 1 and who identified as having type 1 diabetes at wave 3, who took insulin but no non-insulin hypoglycaemic medications at both wave 1 and wave 3 were excluded due to the probability of having type 1 diabetes (*n* = 5).

Participants were grouped by diabetes status at both wave 1 and wave 3 in terms of normoglycaemia, prediabetes and diabetes with those not categorized as having either prediabetes or diabetes classified as having normoglycaemia.

### Vitamin D status

Frozen non-fasting total plasma samples were accessed for the blood biomarker measurements. The concentration of total 25-hydroxyvitamin D (25(OH)D) (including D2 & D3) were quantified by liquid chromatography-mass spectrometry with a validated method (Chromsystems Instruments and Chemicals GmbH; MassChrom 25-hydroxyvitamin D3/D2) in the Biochemistry Department of St James's Hospital (accredited to ISO 15189 standard). Before 25(OH)D was analysed on the mass spectrometer there was extensive clean-up of the samples with steps including protein precipitation, filtration, and further chromatography on the mass spectrometer itself, reducing the risk of potential issues e.g., micro-clots. The quality and accuracy of the method was monitored using internal quality controls, participation in the Vitamin D External Quality Assessment Scheme and the use of the National Institute of Standards and Technology 972 vitamin D standard reference material. The respective inter- and intra-assay coefficients of variation were 5·7% and 4·5%.

Vitamin D deficiency, insufficiency, and sufficiency are defined as <30, 30 to <50 and ≥50 nmol/L, respectively, as per the US Institute of Medicine (IOM) guidelines,^15^ while US Endocrine Society guidelines,^16^ utilise cut-points of <50, 50 to <75 and ≥75 nmol/L. Analysis of both sets of guidelines were completed separately in order to investigate the differences in levels of insufficiency and deficiency. We also combined the IOM and US Endocrine Society guidelines, at <30, 30 to <50, 50 to <75 and ≥75 nmol/L, to present results more efficiently.

### Covariates

Several covariates that have known associations with diabetes were obtained during the TILDA CAPI and health assessments. These included socio-demographic factors such as age, sex, educational level obtained (primary, secondary and third level or higher), in addition to health/behavioural factors such as smoking history, physical activity, statin use (CAPI) and body mass index (BMI) (health assessment).

Smoking history was categorized into non-smoker, light ex-smoker, heavy ex-smoker and current smoker, based on answers to questions in the CAPI on the number of cigarettes smoked per day, number of years smoking and age at which they stopped smoking.

Physical activity (PA) was measured using the International Physical Activity Questionnaire short form, a validated tool to quantify PA, with participants categorized as having low, moderate or high levels of PA.^17^

Statin use was identified by using the World Health Organisation Anatomic Therapeutic Classification codes (C10AA, C10AC, C10AX, C10BA, and C10BX).

Weight was measured to the nearest 0·1 kg (Seca 861 Electronic Scales, Seca Ltd, Birmingham, UK) and height was measured to the nearest 0·01 m (Seca 240 Stadiometer, Seca Ltd, Birmingham, UK). Body mass index (BMI) was calculated using weight and height measurements according to the formula weight (kg) divided by [height (m)]^2^.

The season during which Vitamin D concentrations were sampled was categorized as ‘summer’ (April to September) or ‘winter’ (October to March) depending on the month the blood sample was taken at the health assessment.

Waist circumference was measured to the nearest 0·01 m using a flexible tape measure (Seca Ltd, Birmingham, UK) at the midpoint between the top of the iliac crest and the lower margin of the last palpable rib.

### Statistical analysis and ethics

Stata/MP 14·1 software was used for all statistical analysis (StataCorp, College station, TX). A P-value ≤0·05 was considered statistically significant.

Descriptive statistics were performed for baseline (wave 1) characteristics of TILDA participants including means and 95% confidence intervals (CI) for continuous variable and counts/percent for categorical variables. Data were categorised by diabetes status i.e. normoglycaemia, prediabetes, or diabetes. We used ANOVA and chi-square tests to test for differences between groups for continuous and categorical variables, respectively.

To evaluate the cross-sectional associations between vitamin D concentration (nmol/L) and prevalent diabetes status (normoglycaemia, prediabetes, or diabetes), we utilised multinomial logistic regression to estimate the relative risk ratio (RRR), with 95% CI. Both continuous measurement of 25(OH)D in nmol/L and categories (<30, 30 to <50, 50 to <75 and ≥75 nmol/L) were considered as the primary exposures.

To evaluate associations between baseline vitamin D status and incident disease 4 years later, we utilised data from wave 3 as the outcome. To evaluate incident diabetes, those with prevalent prediabetes and diabetes at baseline were removed from the sample. A multinomial logistic regression model was used to estimate RRR for the association between vitamin D status and diabetes status at wave 3. Both continuous (nmol/L) 25(OH)D and categorical levels using different cut-off points for vitamin D concentration as per US IOM and US Endocrine Society guidelines were examined.

All models were adjusted for age, sex, educational status attained, BMI, smoking history, physical activity, use of statins, and the season in which vitamin D concentration was sampled. All models were repeated using waist circumference (with an interaction effect for sex), in lieu of BMI to investigate if there was a difference in how obesity was measured as a confounder.

Goodness-of-fit of the logistic regression models was examined using Pearson's chi-square test.

The study was reported was done in adherence with STROBE guidelines.^18^

Ethical approval was obtained from the Faculty of Health Science Research Ethics Committee at Trinity College Dublin and written informed consent was obtained from all participants.

### Role of the funding source

The funders of the study had no role in study design, data collection, data analysis, data interpretation, or writing of the report.

All authors had full access to the TILDA dataset used in these analyses. KMC and RAK made the decision to submit the manuscript for publication.

## Results

A total of 5272 TILDA participants who both completed wave 1 and had serum vitamin D measured were included in these analyses. Baseline characteristics included mean age 62·9 years (95% CI: 62·68, 63·17), 53·5% women (95% CI: 52·20, 54·89) and mean vitamin D concentration of 57·1 nmol/L (95% CI: 56·44, 57·82). At wave 1 4612 (87·5%) participants had normoglycaemia, 242 (4·6%) had prediabetes and 418 (7·9%) had diabetes. A comparison of baseline characteristics by diabetes status (Table 1) shows that those classified with diabetes to be older, more likely to be male, and to have completed fewer years of formal education. Vitamin D concentrations were greatest for those with normoglycaemia (58 nmol/L) compared to those with prediabetes (53·8 nmol/L) and diabetes (49·8 nmol/L) (p<0·0001).

**Table 1:** Characteristics of study participants stratified by type 2 diabetes mellitus status at wave 1 (2009-2011) of The Irish Longitudinal Study on Ageing (TILDA).

	Normoglycaemia *n* = 4612	Prediabetes *n* = 242	Diabetes *n* = 418	P value
Age (Years)	62·4 (62·1, 62·7)	66·3 (65·0, 67·7)	66·7 (65·9, 67·7)	<0·0001
Sex (%) Male Female	44.9 (43.5, 46·4) 55.1 (53.6, 56.5)	51·7 (45.3, 57·9) 48.3 (42.1, 54.7)	60·3 (55·5, 64·8) 39.7 (35.1, 44.5)	<0·0001
Education (%) Primary Secondary Third Level +	23·8 (22·6, 25·1) 41·5 (40·1, 42·9) 34·7 (33·4, 36·1)	40·5 (34·5, 46·8) 39·3 (33·3, 45·6) 20·2 (15·6, 25·8)	35·2 (30·7, 39·9) 39·9 (35·4, 44·7) 24·9 (21·0, 29·3)	<0·0001
HbA1c (mmol/mol)	32·0 (31·9, 32·1)	41·1 (40·8, 41·3)	44·3 (43·3, 45·3)	<0·0001
Vitamin D (nmol/L)	58·0 (57·2, 58·7)	53·8 (50·8, 56·8)	49·8 (47·4, 52·2)	<0·0001
BMI (kg/m^2^)	28·2 (28·0, 28·3)	31·2 (30·5, 31·9)	32·2 (31·6, 32·8)	<0·0001
Smoking (%) Non-smoker Light ex-smoker Heavy ex-smoker Smoker	46·5 (45·1, 48·0) 16·5 (15·4, 17·6) 21·6 (20·4, 22·8) 15·4 (14·4, 16·5)	33·5 (27·8, 39·7) 15·7 (11·6, 20·9) 32·2 (26·6, 38·4) 18·6 (14·2, 24·0)	38·3 (33·7, 43·0) 10·3 (7·7, 13·6) 35·2 (30·7, 39·9) 16·3 (13·0, 20·1)	<0·0001
Statin use (%)	31·3 (30·0, 32·7)	43·0 (36·9, 49·3)	66·3 (61·6, 70·6)	<0·0001
Physical activity (%) Low levels Moderate levels High levels	27·9 (26·6, 29·2) 35·5 (34·1, 36·9) 36·6 (35·2, 38·0)	33·9 (28·2, 40·1) 34·3 (28·6, 40·5) 31·8 (26·2, 40·0)	40·9 (36·3, 45·7) 34·9 (30·5, 39·6) 24·2 (20·3, 28·5)	<0·0001
Vitamin D season (Summer, %)	64·5 (63·1, 65·9)	69·0 (62·9, 74·5)	65·3 (60·6, 69·7)	0·35

Note: Data presented as means or proportions with percentages with 95% confidence intervals in brackets. Between group differences were analysed using ANOVA and Chi-Square tests as appropriate. BMI = body mass index.

Table 2 shows the transition of participants’ diabetes status from wave 1 to wave 3 for those who completed both waves of the study. Of those included in the longitudinal analysis, at wave 1 there were 3409 participants (89%) with normoglycaemia, 157 (4%) with prediabetes and 262 (7%) with diabetes. At wave 3, 4 years later, 2992 participants (78%) had normoglycaemia, 499 (13%) had prediabetes and 337 (9%) had diabetes.

**Table 2:** Participants of The Irish Longitudinal Study on Ageing (TILDA) who completed both wave 1 (2009-2011) and wave 3 (2014-2015) stratified by diabetes status at each wave.

	Wave 1 Normoglycaemia n (%)	Wave 1 Prediabetes n (%)	Wave 1 Diabetes n (%)	Wave 1 Total n (%)
**Wave 3 Normoglycaemia n (%)**	2912 (85·4, 97·3)	56 (35·7, 1·9)	24 (9·2, 0·8)	2992 (78·2, 100)
**Wave 3 Prediabetes n (%)**	438 (12·9, 87·8)	50 (31·8, 10·0)	11 (4·2, 2·2)	499 (13·0, 100)
**Wave 3 Diabetes n (%)**	59 (1·7, 17·5)	51 (32·5, 15·1)	227 (86·6, 67·4)	337 (8·8, 100)
**Wave 3 Total n (%)**	3409 (100, 89·1)	157 (100, 4·1)	262 (100, 6·8)	3828 (100, 100)

Note: % expressed as column, row.

**Figure 1:**
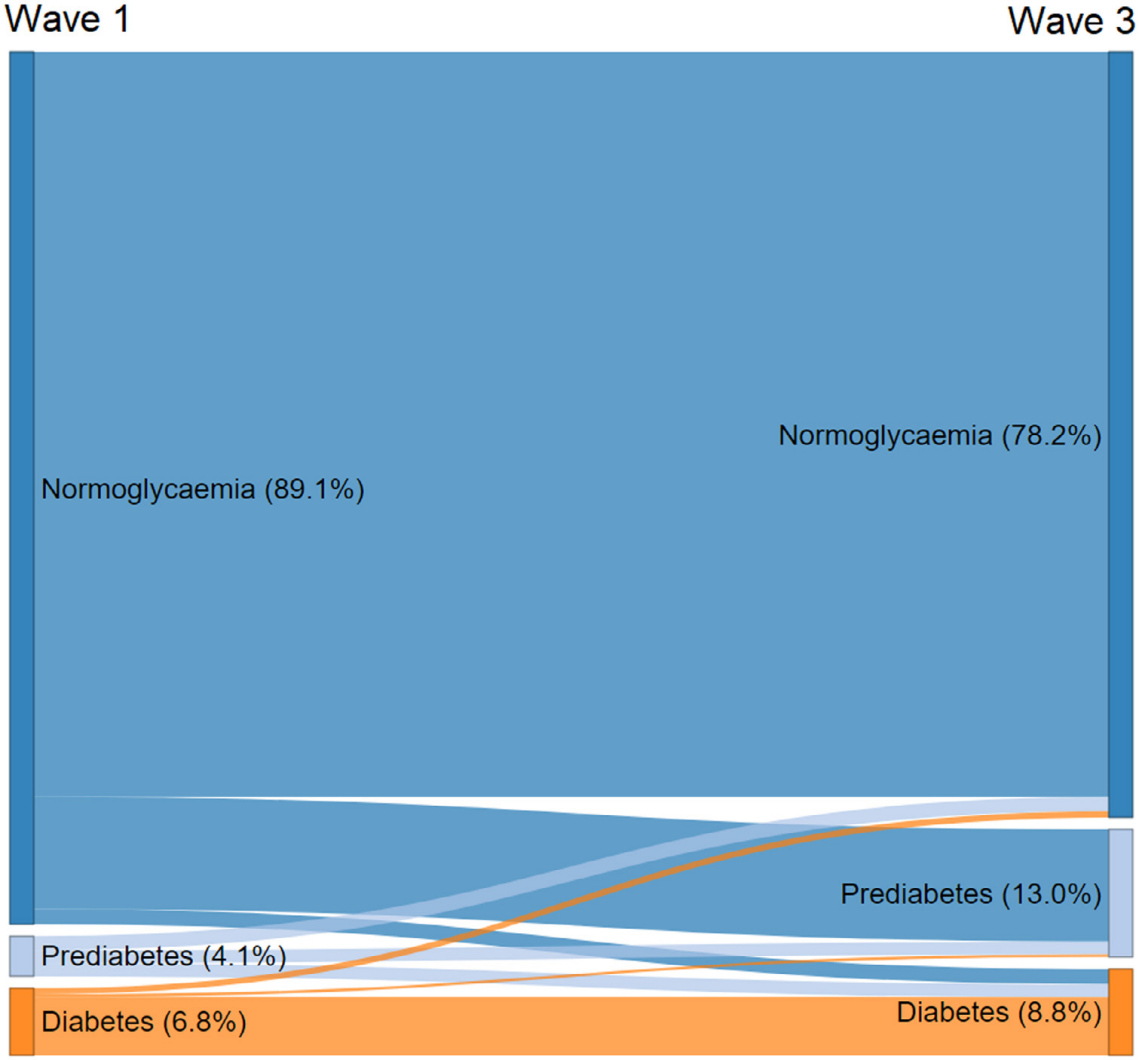
Sankey Plot illustrating participants’ transitions in diabetes status between wave 1 (2009-2011) and wave 3 (2014-2015) of The Irish Longitudinal Study on Ageing (TILDA). Proportions in % for both waves, *n* = 3828.

There were 110 incident cases of diabetes (59 with diabetes at wave 3 from normoglycaemia at wave 1 and 51 with diabetes at wave 3 from prediabetes at wave 1) and 449 incident cases of prediabetes (438 with prediabetes at wave 3 from normoglycaemia at wave 1 and 11 with prediabetes at wave 3 from diabetes at wave 1). While 85% of those with normoglycaemia at wave 1 remained so at wave 3, 12.8% were subsequently classified with prediabetes and 1.7% diabetes 4-years later. Transitions in diabetes status between waves 1 and 3 are illustrated in Figure 1, while baseline characteristics for the 3828 participants included in the longitudinal analysis, stratified by diabetes status at wave 3, are in Table 3. These characteristics are broadly the same as those observed cross-sectionally at wave 1 as detailed in Table 1.

**Table 3:** Baseline characteristics of The Irish Longitudinal Study on Ageing (TLDA) participants, who completed both wave 1 (2009-2011) and wave 3 (2014-2015), stratified by type 2 diabetes mellitus status at wave 3 (*n* = 3828).

	Normoglycaemia *n* = 2992	Prediabetes *n* = 499	Diabetes *n* = 337	*P* value
Age (Years)	61·4 (61·1, 61·7)	64·5 (63·7, 65·3)	65·0 (64·1, 65·9)	<0·0001
Sex (%) Male Female	44·8 (43·0, 46·6) 55.2 (53.4, 57.0)	47·1 (42·7, 51·5) 52.9 (48.5, 57.3)	62·6 (57·3, 67·6) 37.4 (32.4, 42.7)	<0·0001
Education (%) Primary Secondary Third Level +	19·8 (18·4, 21·3) 40·1 (38·3, 41·8) 40·1 (38·4, 41·9)	29·5 (25·7, 33·7) 40·4 (36·1, 44·7) 30·1 (26·2, 34·3)	32·7 (27·8, 37·8) 33·5 (28·7, 38·8) 33·8 (29·0, 39·1)	<0·0001
HbA1c (mmol/mol)	31·6 (31·5, 31·7)	35·2 (35·0, 35·5)	42·6 (41·7, 43·6)	<0·0001
Vitamin D (nmol/L)	59·8 (58·9, 60·7)	53·1 (51·0, 55·1)	52·1 (49·6, 54·5)	<0·0001
BMI (kg/m^2^)	27·8 (27·7, 28·0)	29·7 (29·3, 30·2)	32·0 (31·4, 32·6)	<0·0001
Smoking (%) Non-smoker Light ex-smoker Heavy ex-smoker Smoker	48·3 (46·5, 50·1) 18·4 (17·1, 19·9) 20·4 (18·9, 21·8) 12·9 (11·7, 14·2)	38·7 (34·5, 43·0) 14·2 (11·4, 17·6) 29·3 (25·4, 33·4) 17·8 (14·7, 21·4)	37·7 (32·7, 43·0) 13·1 (9·9, 17·1) 33·2 (28·4, 38·4) 16·0 (12·5, 20·3)	<0·0001
Statin use (%)	29·0 (27·4, 30·6)	42·7 (38·4, 47·1)	57·6 (52·2, 62·7)	<0·0001
Physical activity (%) Low levels Moderate levels High levels	25·5 (23·9, 27·0) 36·3 (34·6, 38·1) 38·2 (36·5, 40·0)	30·5 (26·6, 34·7) 35·8 (31·6, 40·1) 33·7 (29·7, 38·0)	33·3 (28·5, 28·6) 36·7 (31·6, 42·0) 30·0 (25·3, 35·2)	0·002
Vitamin D season (Summer, %)	64·3 (62·6, 66·0)	64·5 (60·1, 68·5)	67·4 (62·1, 72·2)	0·54

Note: Data presented as means or proportions with percentages with 95% confidence intervals in brackets. Between group differences were analysed using ANOVA and Chi-Square tests as appropriate. BMI = body mass index.

Table 4 provides results of the adjusted and unadjusted multinomial logistic regression models for evaluating vitamin D levels with prevalent and incident diabetes. The cross-sectional multinomial logistic regression model (*n* = 5272) showed an association between continuous vitamin D concentration (nmol/L) and likelihood of diabetes with a RRR of 0·99 (95% CI: 0·99, 0·99; *p* = 0·005) in the fully adjusted model. Evaluating vitamin D status in categories showed those at both 30 to <50 nmol/L (RRR 1·62; 95% CI: 1·17, 2·24; *p* = 0·004) and <30 nmol/L (RRR 1·50; 95% CI: 1·03, 2·18; *p* = 0·037) had increased likelihood of prevalent diabetes. No associations between vitamin D status and prevalent prediabetes were found (*p* > 0.25) in the adjusted models.

**Table 4:** Associations between Vitamin D ([25(OH)D] and prevalent prediabetes/diabetes and incident prediabetes/diabetes in participants of the Irish Longitudinal Study on Ageing (TILDA) at wave 1 (2009-11) and wave 3 (2014-15).

		Prediabetes^a^				Diabetes^b^			
		(*n* = 242)				(*n* = 418)			
		Unadjusted Model		Adjusted Model^c^		Unadjusted Model		Adjusted Model^c^	
	Vitamin D	RRR (95% CI)	*P* value	RRR (95% CI)	*P* value	RRR (95% CI)	*P* value	RRR (95% CI)	*P* value
**Prevalent Disease**									
(*n* = 5272)	25(OH)D (nmol/L) (continuous)	0·993 (0·988, 0·999)	0·013	0·999 (0·994, 1·004)	0·79	0·986 (0·981, 0·990)	<0·0001	0·993 (0·988, 0·998)	0·005
	25(OH)D (categorical)								
	≥75 nmol/L	1 (Reference)		1 (Reference)		1 (Reference)		1 (Reference)	
	50-74·9 nmol/L	1·263 (0·866, 1·840)	0·23	1·150 (0·778, 1·698)	0·48	1·198 (0·873, 1·645)	0·26	1·025 (0·731, 1·436)	0·89
	30-49·9 nmol/L	1·599 (1·095, 2·334)	0·015	1·261 (0·850, 1·872)	0·25	2·182 (1·614, 2·951)	<0·0001	1·619 (1·171, 2·238)	0·004
	<30 nmol/L	1·754 (1·127, 2·729)	0·013	1·098 (0·685, 1·759)	0·70	2·544 (1·809, 3·578)	<0·0001	1·500 (1·025, 2·183)	0·037
		**Prediabetes**^a^				**Diabetes**^b^			
		**(*n* = 435)**				**(*n* = 59)**			
**Incident Disease**									
(*n* = 3373)	25(OH)D (nmol/L) (continuous)	0·988 (0·984, 0·993)	<0·0001	0·991 (0·987, 0·996)	<0·0001	0·993 (0·982, 1·004)	0·19	0·997 (0·986, 1·009)	0·61
	25(OH)D (categorical)								
	≥75 nmol/L	1 (Reference)		1 (Reference)		1 (Reference)		1 (Reference)	
	50-74·9 nmol/L	1·173 (0·879, 1·565)	0·28	1·156 (0·858, 1·558)	0·34	1·227 (0·603, 2·494)	0·57	1·021 (0·496, 2·102)	0·96
	30-49·9 nmol/L	1·738 (1·302, 2·320)	<0·0001	1·559 (1·151, 2·112)	0·004	1·260 (0·592, 2·681)	0·55	0·967 (0·445, 2·102)	0·93
	<30 nmol/L	2·019 (1·420, 2·871)	<0·0001	1·619 (1·117, 2·347)	0·011	1·859 (0·775, 4·457)	0·17	1·358 (0·549, 3·357)	0·51

Note: 25(OH)D = 25-hydroxyvitamin D; RRR= relative risk ratio.

a Prediabetes defined as a HbA1c ≥39 mmol/mol and <48 mmol/mol, but not taking any diabetes medications and no self-reported doctor's diagnosis of diabetes.

b Diabetes defined as a self-reported doctor diagnosis of diabetes, use of diabetes medication, or a HbA1c level ≥48 mmol/mol.

c Adjusted for age, sex, educational status attained, body mass index, smoking history, physical activity, use of statins, and the season in which vitamin D level was sampled.

Baseline characteristics for the 3373 participants included in the multinomial logistic regression models evaluating incident disease, stratified by diabetes status at wave 3, are in Table 5.

**Table 5:** Baseline characteristics of The Irish Longitudinal Study on Ageing (TILDA) participants, who completed both wave 1 (2009-2011) and wave 3 (2014-2015), and were included in multinomial logistic regression models evaluating incident disease, stratified by type 2 diabetes mellitus status at wave 3 (*n* = 3373).

	Normoglycaemia *n* = 2879	Prediabetes *n* = 435	Diabetes *n* = 59	*P* value
Age (Years)	61·4 (61·1, 61·7)	64·2 (63·4, 65·1)	62·9 (60·7, 65·2)	<0·0001
Sex (%) Male Female	44·6 (42·8, 46·4) 55.4 (53.6, 57.2)	45·1 (40·4, 49·8) 54.9 (50.2, 59.6)	59·3 (46·3, 71·1) 40.7 (28.9, 53.7)	0.079
Education (%) Primary Secondary Third Level +	19·5 (18·1, 21·0) 40·0 (38·2, 41·7) 40·5 (38·8, 42·3)	29·9 (25·8, 34·4) 39·8 (35·3, 44·5) 30·3 (26·2, 34·8)	30·5 (20·1, 43·4) 37·3 (25·9, 50·3) 32·2 (21·5, 45·2)	<0·0001
HbA1c (mmol/mol)	31·4 (31·3, 31·5)	34·5 (34·3, 34·8)	35·7 (34·0, 37·4)	<0·0001
Vitamin D (nmol/L)	59·9 (59·0, 60·9)	53·3 (51·1, 55·5)	55·5 (49·7, 61·3)	<0·0001
BMI (kg/m^2^)	27·8 (27·6, 27·9)	29·4 (29·0, 29·9)	31·0 (29·8, 32·1)	<0·0001
Smoking (%) Non-smoker Light ex-smoker Heavy ex-smoker Smoker	48·7 (46·8, 50·5) 18·6 (17·3, 20·1) 20·1 (18·7, 21·6) 12·6 (11·4, 13·9)	40·0 (35·5, 44·7) 14·5 (11·5, 18·1) 27·6 (23·6, 32·0) 17·9 (14·6, 21·8)	35·6 (24·4, 48·6) 10·2 (4·6, 21·0) 33·9 (22·9, 46·9) 20·3 (11·9, 32·6)	<0·0001
Statin use (%)	28·8 (27·2, 30·5)	41·6 (37·1, 46·3)	35·6 (24·4, 48·6)	<0·0001
Physical activity (%) Low levels Moderate levels High levels	25·2 (23·7, 26·8) 36·5 (34·7, 38·2) 38·3 (36·6, 40·1)	31·5 (27·3, 36·0) 35·4 (31·0, 40·0) 33·1 (28·8, 37·7)	27·1 (17·3, 39·9) 39·0 (27·4, 52·0) 33·9 (22·9, 46·9)	0·063
Vitamin D season (Summer, %)	64·1 (62·3, 65·8)	63·9 (59·3, 68·3)	71·2 (58·3, 81·3)	0·53

Note: Data presented as means or proportions with percentages and 95% confidence intervals in brackets. Between group differences were analysed using ANOVA and Chi-Square tests as appropriate. BMI = body mass index.

Multinomial logistic regression models evaluating incident disease (*n* = 3373) showed that vitamin D concentrations at wave 1 were not associated with an increased likelihood of developing diabetes at wave 3. RRR per nmol/L increase in vitamin D was 0·99 (95% CI: 0·99, 1·01; *p* = 0·61). However, vitamin D concentrations at wave 1 did have a significant inverse association with the likelihood of having prediabetes at wave 3 with each nmol/L increase in vitamin D having a RRR of 0·99 (95% CI: 0·99, 0·99; p<0·0001).

When vitamin D concentrations were categorized to <30 nmol/L (deficiency), 30 to <50 nmol/L (insufficiency) and ≥50 nmol/L (sufficiency), there were also no significant associations with odds of incident diabetes (*p* > 0·5 in adjusted models). However, there was a 56% increased likelihood of prediabetes for participants whose vitamin D concentrations were insufficient at baseline (RRR 1·56; 95% CI: 1·15, 2·11; *p* = 0·004) and a 62% increased likelihood of prediabetes for those with vitamin D deficiency (RRR 1·62; 95% CI: 1·12, 2·35; *p* = 0·011). No significant associations were found with likelihood of incident diabetes (*p* > 0.5) in adjusted models. All models using waist circumference gave results that were broadly the same as the models using BMI.

**Figure 2:**
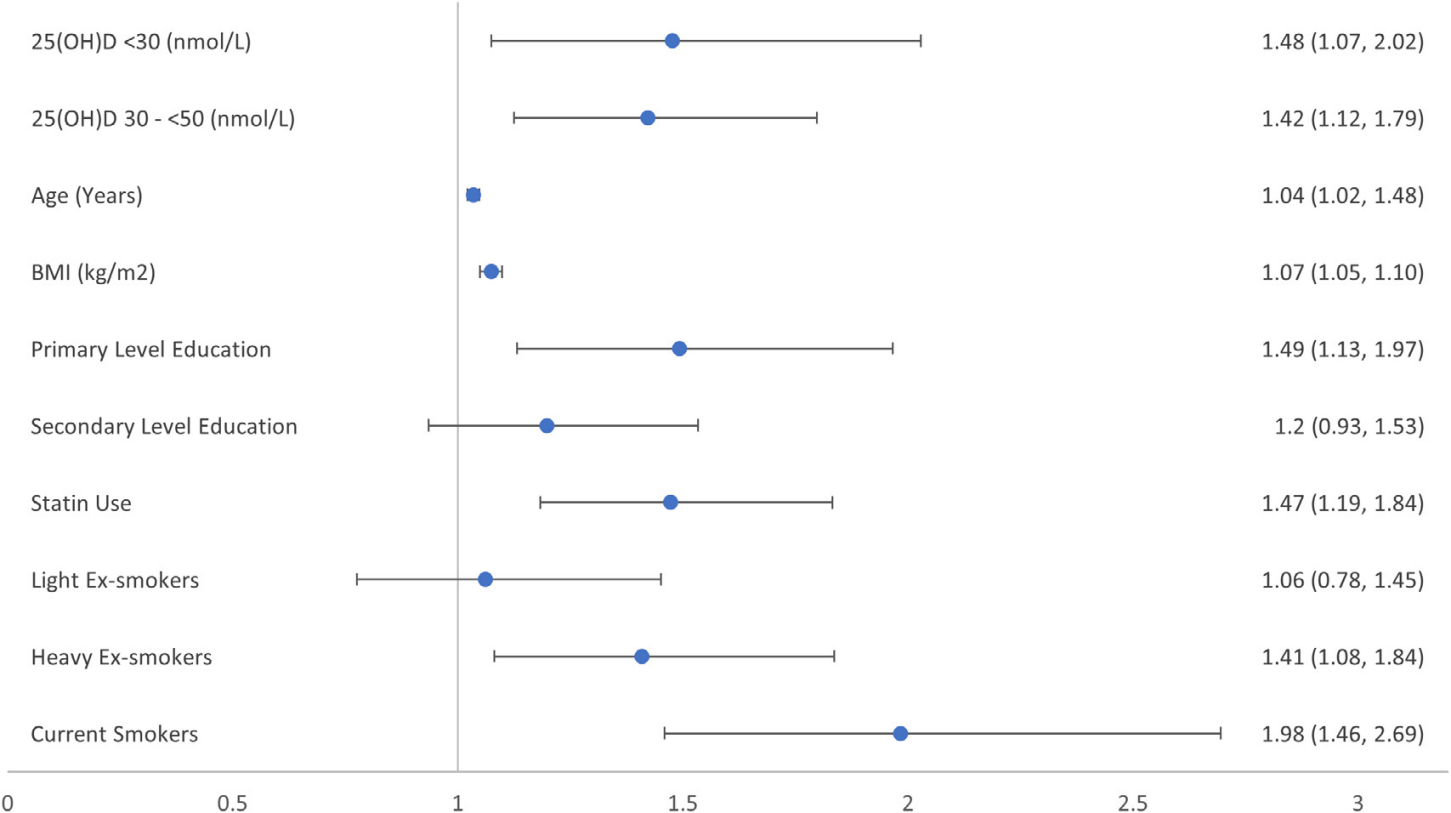
Forest plot of relative risk ratios for Wave 3 prediabetes by vitamin D category at Wave 1 as set out by US Institute of Medicine bone health guidelines. Categorization of The Irish Longitudinal Study on Ageing (TILDA) participants by baseline vitamin D concentrations, to estimate relative risk ratio (95% confidence intervals) for likelihood of incident prediabetes at four-year follow-up in the fully adjusted model; with vitamin D [25(OH)D] concentration ≥50 nmol/L as the reference category for vitamin D. Third level or higher and non-smoker, are the reference category for education, and smoking history respectively. Models were also adjusted for sex, physical activity, and the season during which the vitamin D concentration was sampled, all of which were excluded from the figure as non-significant.

**Figure 3:**
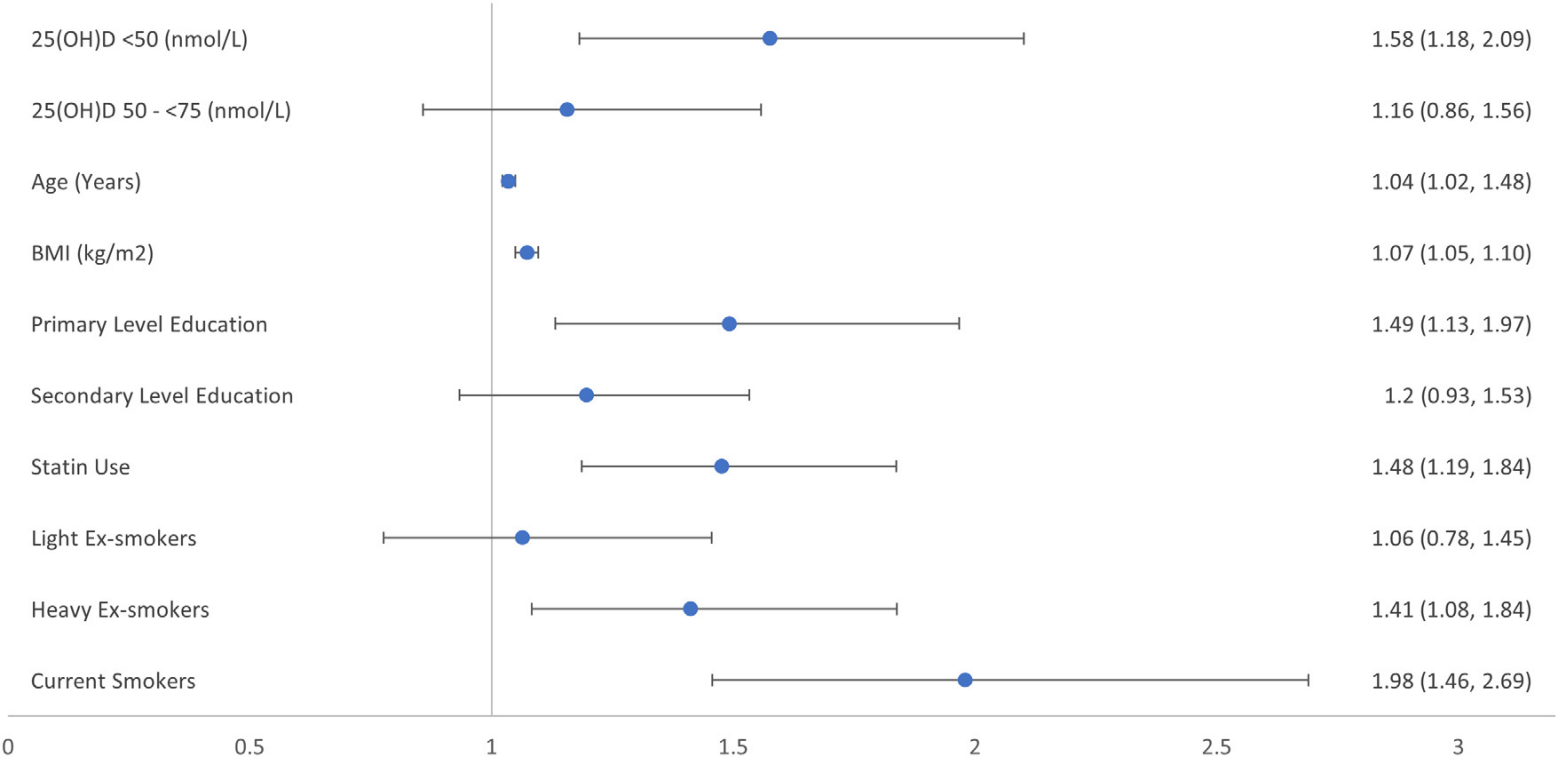
Forest plot of relative risk ratios for Wave 3 prediabetes by vitamin D category at Wave 1 as set out by US Endocrine Society bone health guidelines. Categorization of The Irish Longitudinal Study on Ageing (TILDA) participants by baseline vitamin D concentrations, to estimate relative risk ratio (95% confidence intervals) for likelihood of incident prediabetes at four-year follow-up in fully adjusted model; with vitamin D [25(OH)D] concentration ≥75 nmol/L the reference category for vitamin D. Third level or higher and non-smoker, the reference categories for education, and smoking history respectively. Models were also adjusted for sex, physical activity, and the season during which the vitamin D concentration was sampled, all of which were excluded from the figure as non-significant.

**Figure 4:**
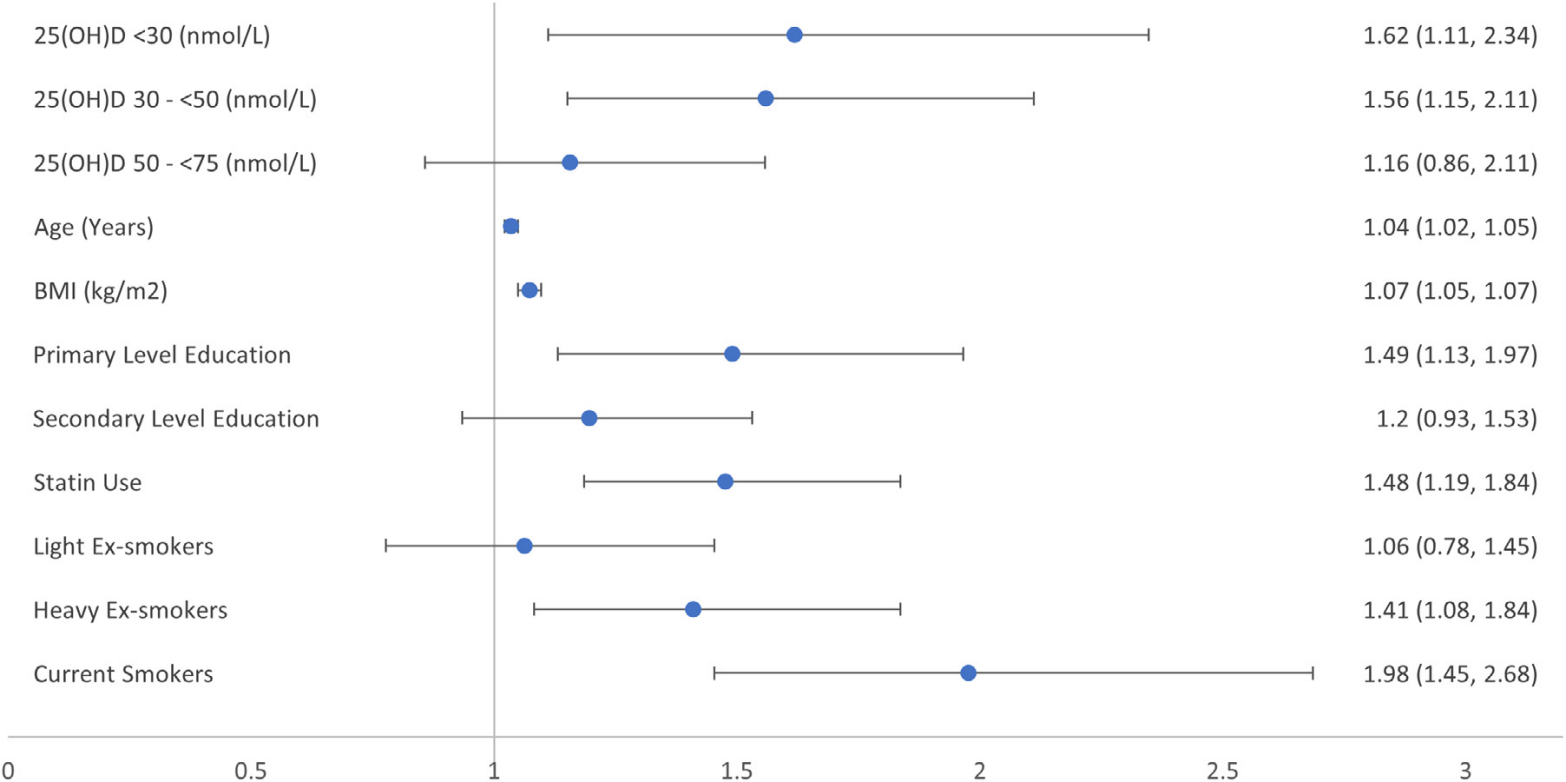
Forest plot of relative risk ratios for Wave 3 prediabetes by vitamin D category at Wave 1 as set out by combination of US Institute of Medicine and US Endocrine Society bone health guidelines. Categorization of The Irish Longitudinal Study on Ageing (TILDA) participants by baseline vitamin D concentrations, to estimate relative risk reduction (95% confidence intervals) for likelihood of incident prediabetes at four-year follow-up in fully adjusted model; with vitamin D [25(OH)D] concentration ≥75 nmol/L the reference category for vitamin D. Third level or higher and non-smoker, the reference categories for education, and smoking history respectively. Models were also adjusted for sex, physical activity, and the season during which the vitamin D concentration was sampled, all of which were excluded from the figure as non-significant.

When we analysed the different levels of deficiency and insufficiency compared to sufficient concentrations of vitamin D as recommended by the US IOM and US Endocrine Society, deficiency increased the likelihood of incident prediabetes 4 years later for both sets of guidelines. Figure 2, Figure 3, Figure 4 provide graphical representation of the increased likelihood of prediabetes using the two different sets of guidelines and a combination of both.

Those included at the outset (*n* = 5332), (those who took part in wave 1 health assessment including blood draws for vitamin D and HbA1c) were younger, attained higher levels of formal education, had lower smoking histories, and were more physically active when compared to those who did not take part in the health assessment (*n* = 2841) and who therefore were not included in the cross-sectional study examining prevalent disease. These findings are in keeping with what has been seen previous studies. Those included in the longitudinal study examining incident disease (*n* = 3828) were younger, attained higher levels of formal education, had lower smoking histories, and were more physically active when compared to those lost to follow-up between wave 1 and wave 3 (*n* = 1504). They also had higher baseline 25(OH)D concentrations, lower HbA1c, lower BMI and were less likely to be taking a statin. These results suggest that those lost to follow-up had poorer health at baseline than those included in the longitudinal study. Characteristics of those not included at baseline and lost to follow-up are included in Supplemental Table 1 and 2 respectively.

## Discussion

In this study of adults aged ≥50 years participating in TILDA, we found significant associations between lower concentrations of vitamin D, as measured by plasma concentrations of 25-hydroxyvitamin D, and both prevalent diabetes and incident prediabetes four years later. These results add to the literature supporting the importance of vitamin D sufficiency at a population level.

This study has shown a cross-sectional association between vitamin D status and diabetes. There was no cross-sectional association observed between vitamin D status and prediabetes. This differs from a recent study using data from a cohort with a similar age profile, although that study used both fasting plasma glucose and results from an oral glucose tolerance test in addition to HbA1c, and consequently had rates of prediabetes (59%) magnitudes higher than observed in this study (4·6%).^19^

In the longitudinal analyses described here, lower baseline concentrations of vitamin D were associated with an increased likelihood of incident prediabetes by wave 3, an interval of four years, with concentrations considered deficient or insufficient by IOM guidelines (i.e. <50 nmol/L) being 57% more likely to develop prediabetes compared to those with concentrations of ≥75 nmol/L. Those with insufficient concentrations, as per the IOM guidelines (i.e. 30 to <50 nmol/L), had a 42% increased likelihood of incident prediabetes when compared to those with sufficient concentrations (i.e. ≥50 nmol/L) and a 55% increased likelihood of incident prediabetes when compared to those with concentrations ≥75 nmol/L. This finding would call into question any recommendation that state it is acceptable to have vitamin D concentrations at anything less than 50 nmol/L. There was limited ability to examine the association between vitamin D status at wave 1 and the likelihood of incident diabetes by wave 3, as only 110 participants (59 from normoglycaemia and 51 from prediabetes) developed diabetes between these waves, with only 59 included in our models when those with baseline prediabetes were excluded.

There have been several trials where vitamin D supplementation has been examined to prevent diabetes,^20^, ^21^, ^22^ and have not shown that it reduced the risk of diabetes. These studies have largely included those at highest risk of developing diabetes, namely those already with prediabetes, whereas this longitudinal observational study included development of prediabetes as an outcome. The participants in these interventional studies have also tended to have baseline vitamin D concentrations far higher, with very low levels of deficiency (<30 nmol/L) and low levels of insufficiency (30 to <50 nmol/L) than what has been shown in observational studies. For example, the D2d study,^20^ a randomised control trial with over 2000 participants, had mean vitamin D concentrations of 70 nmol/L, with 78% having concentrations ≥50 nmol/L and only 4·3% having concentrations <30 nmol/L. This compares to a mean concentration of 58nmol/L, with 60% having concentrations ≥50 nmol/L and 11·5% having concentrations <30 nmol/L of the 3828 participants included in this study. 22% of participants in the D2d study had vitamin D concentrations <50 nmol/L,^20^ which is far less than what has been found in observational studies such as TILDA where 42·5% had vitamin D concentrations of <50 nmol/L,^9^ or the Longitudinal Ageing Study Amsterdam where half had vitamin D concentrations <50 nmol/L.^23^ While the hazard ratio for vitamin D supplementation in the D2d study was 0·88 (95% CI 0·75, 1·04, *p* = 0·12), when compared to placebo, a post hoc analysis of data for participants with baseline vitamin D concentrations of <30 nmol/L showed a hazard ratio of 0·38 (95% CI 0·18, 0·80) for those in the vitamin D supplement group.^20^ Another criticism of some of the trials carried out to date has been that they may have been underpowered to test for the effect size they were examining.^21^ While this study did not show that low vitamin D concentrations at wave 1 were associated with an increased likelihood of incident diabetes at wave 3, as mentioned previously, only 110 participants developed diabetes between these waves, so these models too may have been underpowered. The results with prediabetes, however, suggest that longer follow-up of TILDA may provide the number of incident diabetes cases to potentially show significant associations in the future.

This study has shown that 12·8% (438 of 3409) of those with normoglycaemia at wave 1 went on to develop prediabetes at wave 3, an average of 3·2% per annum (pa). The rate of progression from prediabetes to diabetes is 5-10% pa with up to 70% of those with prediabetes developing diabetes eventually.^24^^,^^25^ In our study 32·5% (51 of 157) of those with prediabetes at wave 1 went on to develop diabetes at wave 3, an average rate of over 8% pa. This differs from a recent study by Rooney *et al.* that showed that only 9% of those with prediabetes progressed to diabetes within a median period of five years, with regression to normoglycaemia or death both being more frequent at 13% and 19% respectively.^26^ A significant difference between that study and this one is that the 3828 included here have a mean age of 62·1 while the 3412 included in the Rooney *et al.* study were older with a mean age of 75·6. That study did not examine vitamin D and there are quite likely to be several differences between the two cohorts. If age was the main difference, it would suggest that prediabetes is more likely to progress to diabetes in younger people than older and may suggest a difference in the underlying pathophysiology. Participant selection for potential future trials may need to reflect this.

This study has shown that baseline vitamin D concentrations <50 nmol/L associate with an increased likelihood of incident prediabetes, whereas there was no significant difference in association between those with vitamin D concentrations of 50 to <75 nmol/L and ≥75 nmol/L. That suggests that those with vitamin D concentrations <50 nmol/L, deficient or insufficient, as per IOM guidelines, stand to benefit most from bringing their concentrations ≥50 nmol/L. This should be considered when reviewing the appropriateness of populations chosen in many of the interventional trials given the participants already had largely sufficient vitamin D concentrations. As mentioned previously some of the studies may have been underpowered and duration of follow-up too short.

It has been estimated that while 9 micrograms per day of vitamin D would maintain vitamin D concentrations >25 nmol/L in >97·5% of adults during winter time in Ireland, 28 micrograms/day of vitamin D would be required to maintain vitamin D concentrations >50 nmol/L,^27^ which is significantly higher than that generally consumed by adult populations.^28^ 25(OH)D concentrations increased from a mean of 69 nmol/L at baseline to a mean of 135 nmol/L among participants receiving 100 micrograms per day of vitamin D over a 24 month period in the D2d study.^20^ Mandatory fortification would be most beneficial to those likely to have vitamin D concentrations <50 nmol/L, those who do not take in enough vitamin D through their diet, via supplementation or via cutaneous synthesis during summer months.

Glycaemia is a spectrum, with the degree of hyperglycaemia associated with different complications varying depending on the complication without consistent thresholds. Diagnostic criteria for diabetes are based on the degree of hyperglycaemia associated with increased risk of diabetic retinopathy,^29^ whereas lower levels of hyperglycaemia, that would fall within the prediabetes diagnostic criteria, are associated with increased risk of diabetic neuropathy,^30^ and cardiovascular disease.^31^ Therefore, improving hyperglycaemia and reducing the incidence of prediabetes would be of significant benefit to potential underlying disease states.

Apart from the association between vitamin D concentrations and likelihood of incident prediabetes, our regression models demonstrated that each 1-year increase in age increased the likelihood of developing prediabetes by 3%. Also, each kg/m^2^ increase in BMI increased the likelihood of incident prediabetes by 7%. It has long been considered that 25(OH)D concentrations were lower in those overweight and obese, with blunted increases to pharmacological doses of vitamin D, due to sequestration of vitamin D, a fat-soluble vitamin, in adipose tissue. More recently obesity has been shown to decrease hepatic 25-hydroxylase activity causing low 25(OH)D concentrations due to decreased expression of CYP2R1, the principal hepatic vitamin D 25-hydroxylase, while the vitamin D endocrine system is now felt to be far more complex than originally thought.^32^^,^^33^ Having attained an education to third level or higher was associated with a reduction in the likelihood of incident prediabetes by 33%, when compared to attaining an education to primary school level. Being a current smoker was associated with a 98% increased likelihood of incident prediabetes, and heavy ex-smokers a 41% increased likelihood, when compared to non-smokers. Smoking is known to be a risk factor for incident diabetes with the benefits of cessation only apparent at least 5 years after stopping smoking.^34^ Taking a statin regularly was associated with a 48% increased likelihood of incident prediabetes. Statin therapy is associated with a small increased risk of incident diabetes,^35^ but its association with prediabetes has not been well characterised. Sex and levels of physical activity were not associated with increased likelihood of incident prediabetes over this 4-year follow-up period.

This study has a number of strengths including large sample size, high quality data collection which was standardised across waves, and the ability to conduct longitudinal analyses.

In terms of limitations of this study, while there is a strong rationale for the study hypothesis from a biological point of view, low vitamin D concentrations may be more of a surrogate marker for poor health or broader socio-economic variables that cannot be fully controlled for, such as impact on vitamin D concentrations due to regular outdoor activity and dietary choices. Vitamin D concentrations may change with time and this study only considers baseline vitamin D status. This could be considered a limitation over a 4-year follow-up period; however, it has clinical relevance from a practical level in that clinicians often only have isolated laboratory results from which to make risk assessments and management plans for their patients. The length of the follow-up period could be considered a weakness – 4 years is a reasonably short period of time when the development of insulin resistance, prediabetes and diabetes can take over 10 years, however, given the significant effect size observed over only a 4-year follow-up period makes the findings even more meaningful. Genetic variations of vitamin D-binding protein and VDR have been associated with insulin resistance in different populations independent of obesity.^12^^,^^36^ This study does not consider potential genetic differences in vitamin D-binding protein and VDR within the population studied.

As with any longitudinal study, full engagement and follow-up within TILDA is imperfect, due to several reasons such as morbidity, mortality, disengagement and change in life circumstances. 2841 (34·7%) participants at wave 1 were not included due to not taking part in the health assessment and therefore missing data with regards to HbA1c and 25(OH)D concentration. A further 1504 (28·2%) of the 5332 participants included at baseline were lost to follow-up at wave 3. Characteristics of those not included at baseline and lost to follow-up are included in Supplemental Table 1 and 2 respectively, to allow an understanding of the differences between these groups.

This study assumes that the pathophysiology of developing diabetes is uniform when it may differ between individuals e.g. age-related insulin deficiency versus insulin resistance versus other causes such as haemochromatosis. Data relating to participants’ family history of diabetes was not available so genetic predisposition to developing hyperglycaemia could not be considered in the analyses. While an accepted diagnostic test for diabetes and prediabetes, using a single HbA1c alone as a measure to diagnose diabetes and/or prediabetes is imperfect and does not account for factors that may have an impact on haemoglobin glycation independent of glycaemia, such as anaemia. HbA1c alone is also not as comprehensive as also having fasting plasma glucose levels and/or oral glucose tolerance test results. A measurement of insulin resistance such as HOMA-IR (Homeostatic Model Assessment for Insulin Resistance) would have added a lot of value to the study, however, unfortunately HbA1c is currently the only available measurement to estimate insulin resistance in the TILDA study. Fasting insulin and fasting glucose levels were not measured, and in fact no fasting blood samples were taken due to the health assessments being carried out throughout the day, with many participants not having their blood draw done until evening time. It was not felt to be responsible to ask participants, many of whom were at an advanced age and/or with numerous co-morbidities, to fast for such prolonged periods.

In conclusion, the reduction in likelihood for developing prediabetes was greatest for higher concentrations of vitamin D, as measured by 25-hydroxyvitamin D, with concentrations of ≥75 nmol/L associated with having over a third less likelihood of developing prediabetes compared to those with concentrations <50 nmol/L. There was no significant reduction in likelihood of developing prediabetes for concentrations of 30 to <50 nmol/L but that concentration was associated with a 42% increased likelihood of incident prediabetes compared to those with sufficient levels, suggesting that the benefit of higher vitamin D concentration become significant from 50 nmol/L, which are also the concentrations considered sufficient from a bone health point of view.^16^ Given that vitamin D concentrations of ≥75 nmol/L were associated with a reduced likelihood of developing prediabetes by over a third in a four-year period, then it is reasonable to conclude that optimising vitamin D status may significantly reduce, or at least delay, the numbers progressing to diabetes and the underlying complications associated with chronic hyperglycaemia.

## Contributors

K.M.C., E.L. and R.A.K. conceived the study. K.M.C., E.L., A.O., A.F. and M.H. were responsible for methodology, validation and data curation. K.M.C., C.W., A.F., and B.H. were responsible for statistical analysis. K.M.C. and E.L. had full access to the data and can verify the data presented. All authors had full access to the data, critically revised the manuscript content and gave final approval for the version to be published. All authors accept responsibility to submit for publication.

## Data sharing statement

TILDA data can be accessed via the Irish Social Science Data Archive (www.ucd.ie/issda). The publicly accessible dataset files are hosted by the Irish Social Science Data Archive based in University College Dublin, and the Interuniversity Consortium for Political and Social Research (ICPSR) based in the University of Michigan. Researchers wishing to access the data must complete a request form, available on either the ISSDA or ICPSR website.

## Declaration of interests

All authors declare no competing interests.

## Appendix Supplementary materials

Image, application 1
