# Supplementary material for: Association between vitamin D deficiency and the risk of prevalent type 2 diabetes and incident prediabetes: A prospective cohort study using data from The Irish Longitudinal Study on Ageing (TILDA)
Source: eClinicalMedicine. 2022 Sep 17;53:101654. doi: 10.1016/j.eclinm.2022.101654 (PMC9486023; doi:10.1016/j.eclinm.2022.101654)
Supplement: Supplementary file 1 [file mmc1.pdf]

Supplemental Table 1: Baseline characteristics of The Irish Longitudinal Study on Ageing (TILDA) participants stratified by inclusion status at wave 1 (2009-2011) (n=8173)

	**Not included n=2841**	**Included n=5332**	**P value**
Age (Years)	65·5 (65·1, 65·9)	62·9 (62·7, 63·2)	p<0·0001
Sex (%)			
Male	44·5 (42·7, 46·3)	46·5 (45·1, 47·8)	p=0·079
Female	55·5 (53·7, 57·3)	53·5 (52·1, 54·8)	
Education (%)			
Primary	40·3 (38·5, 42·1)	25·5 (24·4, 26·7)	
Secondary	37·5 (35·8, 39·3)	41·2 (39·9, 42·6)	p<0·0001
Third Level +	22·2 (20·7, 23·8)	33·3 (32·0, 34·5)	
Smoking (%)			
Non-smoker	40·7 (38·9, 42·5)	45·2 (43·9, 46·6)	
Light ex-smoker	12·4 (11·3, 13·7)	16·0 (15·0, 17·0)	p<0·0001
Heavy ex-smoker	23·8 (22·3, 25·4)	23·2 (22·0, 24·3)	
Smoker	23·1 (21·6, 24·7)	15·6 (14·7, 16·6)	
Physical activity (%)			
Low levels	37·6 (35·8, 39·4)	29·1 (27·9, 30·4)	
Moderate levels	32·4 (30·6, 34·1)	35·5 (34·2, 36·8)	p<0·0001
High levels	30·0 (28·4, 31·8)	35·4 (34·1, 36·7)	

Note: Data presented as means or proportions with percentages and 95% confidence intervals in brackets· Between group differences were analysed using ANOVA and Chi-Square tests as appropriate· BMI = body mass index

Supplemental Table 2: Baseline characteristics of The Irish Longitudinal Study on Ageing (TILDA) participants stratified by loss to follow-up status at wave 3 (2014-2015) (n=5332)

	**Lost to follow-up n=1504**	**Not lost to follow-up n=3828**	**P value**
Age (Years)	65·0 (64·5, 65·5)	62·1 (61·9, 62·4)	p<0·0001
Sex (%)			
Male	46·1 (43·6, 48·7)	46·7 (45·1, 48·2)	p=0·74
Female	53·9 (51·3, 56·4)	53·3 (51·8, 54·9)	
Education (%)			
Primary	31·2 (27·9, 34·7)	22·2 (20·9, 23·5)	
Secondary	40·1 (36·6, 43·7)	39·5 (38·0, 41·1)	p<0·0001
Third Level +	28·7 (25·5, 32·1)	38·3 (36·8, 39·8)	
HbA1c (mmol/mol)	34·2 (33·9, 34·6)	33·0 (32·9, 33·2)	p<0·0001
Vitamin D (nmol/L)	54·2 (52·9, 55·5)	58·3 (57·5, 59·1)	p<0·0001
BMI (kg/m^2)	29·1 (28·8, 29·3)	28·4 (28·3, 28·6)	p<0·0001
Smoking (%)			
Non-smoker	43·0 (40·5, 45·5)	46·1 (44·5, 47·7)	
Light ex-smoker	12·1 (10·5, 13·8)	17·4 (16·3, 18·7)	p<0·0001
Heavy ex-smoker	24·7 (22·6, 26·9)	22·7 (21·4, 24·0)	
Smoker	20·3 (18·3, 22·4)	13·8 (12·8, 14·9)	
Statin use (%)	37·7 (35·3, 40·2)	33·3 (31·8, 34·8)	p=0·002
Physical activity (%)			
Low levels	35·6 (33·2, 38·1)	26·8 (25·4, 28·2)	
Moderate levels	33·1 (30·7, 35·5)	36·3 (34·8, 37·8)	p<0·0001
High levels	31·3 (29·0, 33·7)	36·9 (35·4, 38·5)	
Vitamin D season (Summer, %)	65·1 (62·7, 67·5)	64·6 (63·1, 66·1)	p=0·704

Note: Data presented as means or proportions with percentages and 95% confidence intervals in brackets· Between group differences were analysed using ANOVA and Chi-Square tests as appropriate· BMI = body mass index

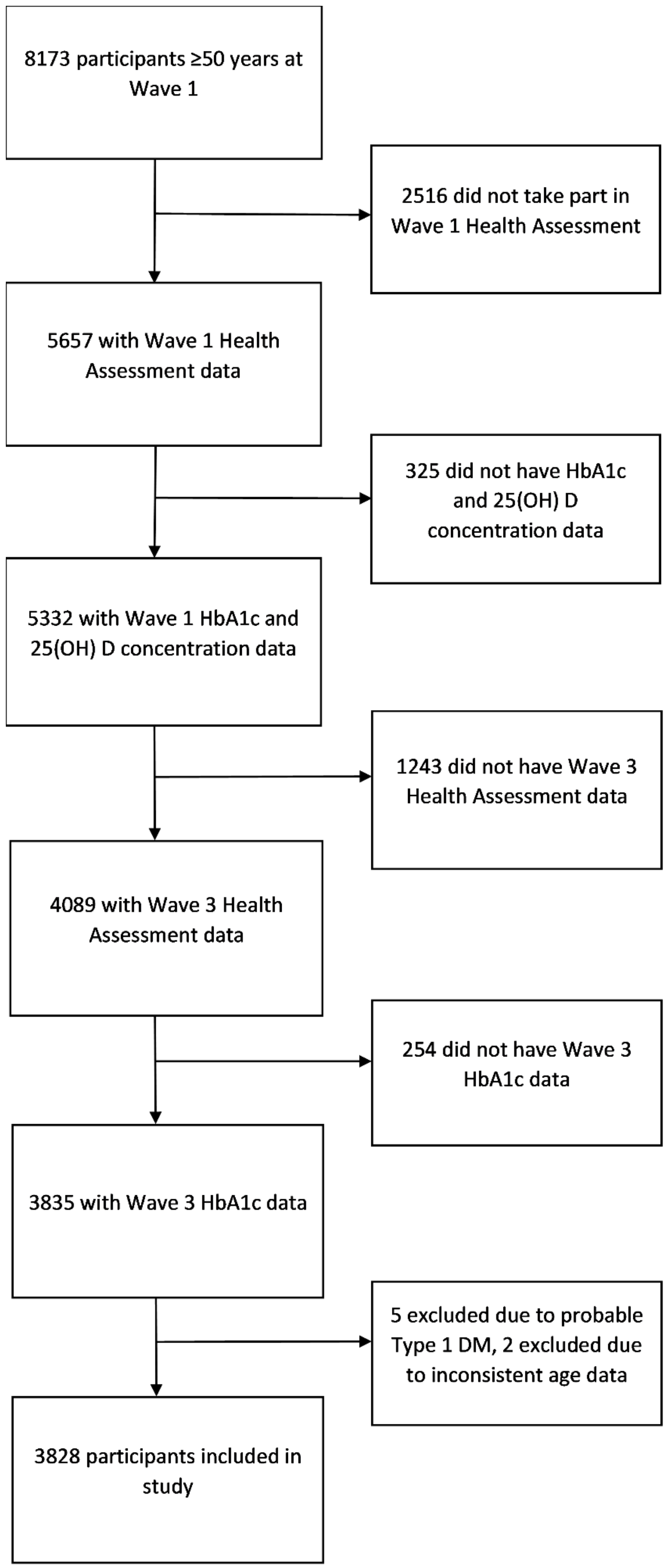

Supplemental Figure 1: Flowchart of participant inclusion criteria for study
